# The clinical status and survival in elderly dialysis: example of the oldest region of France

**DOI:** 10.1186/1471-2369-14-131

**Published:** 2013-06-25

**Authors:** Florence Glaudet, Carine Hottelart, Julien Allard, Vincent Allot, Frédérique Bocquentin, Rémy Boudet, Béatrice Champtiaux, Jean Pierre Charmes, Monica Ciobotaru, Zara Dickson, Marie Essig, Philippe Honoré, Céline Lacour, Christian Lagarde, Maria Manescu, Pierre Peyronnet, Jean Michel Poux, Jean Philippe Rerolle, Michel Rincé, Cécile Couchoud, Jean Claude Aldigier

**Affiliations:** 1 Nephrology Unit, Limoges University Hospital, Limoges, France; 2 ALURAD (Limousine Association for the use of the artificial kidney at home, Limoges, France; 3 Nephrology Unit, Brive Hospital, Brive, France; 4 ALURAD (Limousine Association for the use of the artificial kidney at home, Brive, France; 5 REIN registry, Biomedecine Agency, La Pleine-Saint Denis, France

**Keywords:** Co-morbidity, Elderly, End stage renal disease, Outcome, Survival

## Abstract

**Background:**

The number of elderly (≥75 years) patients with end-stage renal disease (ESRD) has increased markedly, including in the Limousin region, which has the oldest population in France. We retrospectively compared outcomes in elderly and non-elderly ESRD patients who started dialysis during two time periods.

**Methods:**

Baseline clinical characteristics, care, and survival rates were assessed in 557 ESRD patients aged ≥75 and <75 years who started dialysis in 2002–2004 and 2005–2007. Survival curves and Cox proportional hazards model were used to assess survival and factors associated with survival.

**Results:**

Of the 557 patients, 343 and 214 were <75 years and ≥75 years, respectively. Dialysis was started in 2002–2004 and 2005–2007 by 197 and 146 patients <75 years, respectively, and by 96 and 118 patients ≥75 years, respectively. Median age (73.4 years [interquartile range [IQR] 61.7-79.5 years] *vs* 69.5 years [IQR 57.4-77.4 years] p = 0.001) and the proportion aged ≥75 years (44.7% *vs* 32.8%, p = 0.004) were significantly higher in 2005–2007 than in 2002–2004. Improved initial status during 2005–2007 was observed only in patients ≥75 years, with a decrease in some co-morbidities, improved walking and better preparation for dialysis. Mortality rates were significantly lower in 2005–2007 than in 2002–2004 (hazard ratio 0.81, 95% confidence interval 0.69-0.95; p = 0.008), with the difference due to factors associated with clinical status and care.

**Conclusions:**

Improved initial clinical status and better preparation for dialysis, accompanied by increased survival, were observed for patients ≥75 years who started dialysis more recently, perhaps because of early referral to a nephrologist.

## Background

Over the past 10 years, all countries in Europe have experienced increases in the rate of elderly people beginning dialysis [1,2]. For example, 37.9% of patients in France who started dialysis in 2009 were older than 75 years [3]. Identifying the characteristics of this elderly population is important in adapting policies for appropriate care and in estimating future needs by renal care organisation. The higher incidence of end-stage renal disease (ESRD) may be due in large part to the ageing of the general population and the broader access to dialysis therapy among older persons with renal failure [4,5]. Multivariate analysis has shown that increasing age is significantly predictive of survival in elderly patients on dialysis [6-9]. Other studies have shown that the excess mortality observed in people on dialysis compared with the general population was less apparent in older than in younger patients [10,11].

Limited data are available on age-related differences in the clinical status of patients on dialysis and their trends over time. A study comparing differences between countries in haemodialysis patients according to age observed country-related differences in management practices of elderly individuals, but did not assess changes in management practices over time [12].

Limousin is a region in France included in the “REIN” registry, which provides quality controlled data about patients with ESRD. In 2010, 44.3% of individuals on dialysis in Limousin were over 75 years old, compared with 39.2% of individuals throughout the rest of France. To assess the impacts of age and management practices over time in patients with ESRD, we analysed the baseline clinical characteristics and care of all patients, aged <75 and ≥75 years, who started dialysis between in 2002–2004 and 2005–2007. Survival on dialysis was compared in patients who started dialysis in these two time periods, and factors associated with improved survival in elderly patients were analysed.

## Methods

### Population

The population of Limousin consisted of 718,716 residents in 2002 and 737,001 in 2007. All patients living in the Limousin region and who had started long-term dialysis between January 1, 2002, and December 31, 2007 were included in the study. These 557 patients were divided into four groups, by age (<75 and ≥75 years) and period starting dialysis (2002–2004 and 2005–2007). Treatment modalities for haemodialysis included three haemodialysis centres located in health facilities with physicians always available (in-centre haemodialysis), three haemodialysis centres without a physician always available (out-centre haemodialysis), three self-care units and home haemodialysis. Peritoneal dialysis (PD) was either continuous ambulatory or automated.

### Data source

The REIN design has been described in detail [13]. The data included in this study were released from the REIN registry data under the responsibility of the authors, and with the approval of the Regional Committee in Limousin. Data gathered for this study included patient demographics, co-morbidities, severe disability, mobility, nutritional status, renal function, anaemia status, and dialysis modality. Primary renal diseases and renal biopsy before dialysis were included. The eight co-morbidities assessed were diabetes, chronic respiratory disease, heart failure, cerebrovascular disease, peripheral vascular disease, arrhythmia, coronary vascular disease and active malignancy. The two vascular risk factors analysed were hypertension and smoker/ex-smoker.

Handicaps included severe disabilities (severely impaired vision, amputation, hemiplegia and paraplegia) and severe behavioural disorders (dementia, psychosis, and severe neurosis that may affect patient dependence or compliance with treatment). Mobility was classified into three groups according to patient dependence. Serum albumin concentrations were measured and body mass index (BMI) was calculated as weight (kg)/square of height (m). Data on mobility and BMI were missing for <5% of patients.

An urgent first dialysis was defined as its immediate performance after evaluation by a nephrologist due to risk of vital status, poorly tolerated anaemia, pericarditis or uremic confusion of origin.

Renal function was assessed as estimated glomerular filtration rate (eGFR), which was calculated using the modification of diet in renal disease (MDRD) formula. Pre-dialysis anaemia and first treatment modalities were also determined.

### Outcome

All patients were monitored for 4 years after their first dialysis. Major events included renal transplantation, changes in place of dialysis, transient recovery of renal function and death. Vital status was checked monthly, so that event records can be considered exhaustive.

### Statistical analysis

The annual incidence rates were age-standardized using as reference the estimate of the metropolitan French population for each year considered.

The impact of demographic factors, age structure and renal disease risk on ESRD incidence was analysed using the RiskDiff web tool [14], established as described [15].

Patients’ baseline characteristics were compared using the chi-square test and Student’s t-test.

In analysing survival, patients were censored if they underwent renal transplantation during the follow-up period or were still alive and on dialysis at the end of follow-up. The chi-square test was used to compare the proportion of deaths four years after the start of dialysis. Four-year survival analysis consisted of three steps. First, the Kaplan-Meier method was used to estimate patient survival by stratum (age group and study period), with survival curves compared using log rank tests. Age groups showing significantly different mortality outcomes during the two treatment times were analysed for factors associated with this difference. Thus, in the second step for this group, a Cox proportional hazard model was used to analyse risk factors associated with death during the first 48 months (univariate analysis), regardless of the dialysis initiation period. Finally, in order to analyse the association between study period and mortality, five Cox proportional hazard models were fitted with sequential adjustments for confounding variables (multivariate analysis). Model-1 was adjusted for age and gender; Model-2 was Model-1 plus adjustments for co-morbidities associated with survival during the previous step; Model-3 was Model-1 plus adjustments for mobility; model-4 was Model-1 plus adjustments for initial condition at the start of dialysis; and Model 5 included all variables used in the other models.

Hazard ratios [HRs] and their 95% confidence intervals [CIs] were calculated. All data were analysed using JMP 6 (SAS Institute, Cary, NC). A p-value <0.05 was defined as statistically significant.

## Results

### Baseline characteristics

During the study period, 557 patients underwent dialysis, including 293 in 2002–2004 and 264 in 2005–2007. Of the patients who started dialysis in 2002–2004, 197 were < 75 and 96 were ≥75 years old; In 2005–2007, 146 were <75 and 118 were ≥75 years old. Median age of cohorts were 69.5 years [IQR 57.4-77.4 years] in 2002–2004 and 73.4 years [IQR 61.7-79.5 years] in 2005–2007 (p = 0.001). Among the patients ≥75 years old, median age was 79.9 years [IQR 77.5-82.9 years] in 2002–2004 and 80.3 years [IQR 77.4-83.3 years] in 2005–2007 (p = NS).

**Figure 1:**
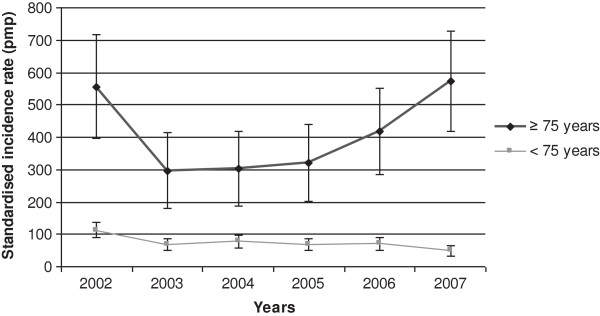
Standardized incidence by age group between 2002 and 2007.

Figure 1 shows the change in incidence by age group. The older age group showed an increase in incidence of ESRD between 2003 and 2007, especially after 2006. The incidence was high (385 per million [IQR 254–516 per million]) in 2002–2004, but was even higher (437 per million [IQR 301–573 per million] in 2005–2007). The incidence of ESRD in patients ≥ 75 years increased from 397 per million [IQR 262–532 per million] in 2002–2004 to 457 per million [IQR 313–597 per million] in 2005–2007.

**Figure 2:**
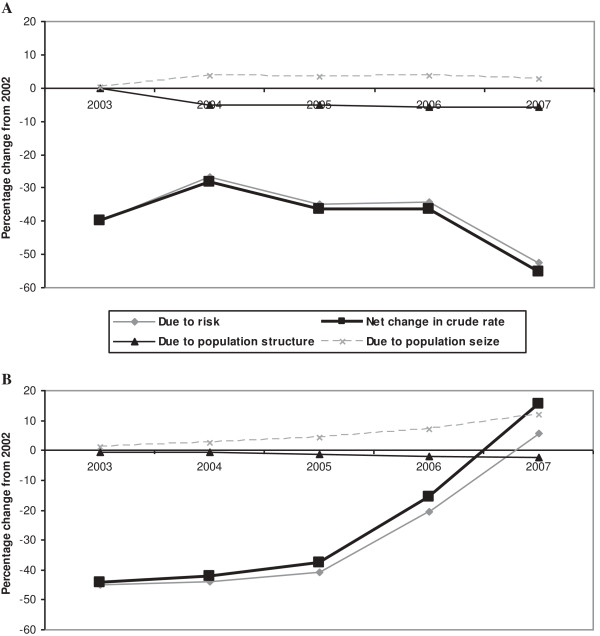
**Differences in incidence from 2003 to 2007 compared to the baseline year 2002.** Legend of Figure 2: **A**. <75 years; **B**. ≥75 years.

Figure 2 shows the real contribution of kidney disease to the change in ESRD incidence over time, taking into account changes in the general population between 2002 and 2007. The increased incidence in ESRD for patients ≥75 years between 2003 and 2007 was associated with an increase in the proportion of the elderly population, but primarily to the increase in kidney disease risk. In the younger population, those aged <75 years, the decreased incidence of ESRD was due mainly to a lower risk of kidney disease.

Table 1 shows the baseline characteristics of patients. In patients aged <75 years, the percentages with cerebrovascular disease, peripheral vascular disease and arrhythmia were significantly higher in 2005–2007 than in 2002–2004. The percentage of patients aged <44 years was lower in 2005–2007. When only patients aged 45–74 years were assessed, there were also significant increases over time in these three co-morbidities. The first treatment modality has changed significantly over time, with a decrease in the number of patients undergoing peritoneal dialysis and an increase in those undergoing haemodialysis with fistula (Table 2).

**Table 1:** Patient clinical baseline characteristics at dialysis initiation according to age and period

	**<75 yr**			**≥75 yr**			
	**2002-2004**	**2005-2007**	**P1**	**2002-2004**	**2005-2007**	**P2**	**P3**
	**(n = 197)**	**(n = 146)**		**(n = 96)**	**(n = 118)**		
	**%**	**%**		**%**	**%**		
*Age*			0.05			NS	
0-19	3(1.5)	1(0.7)					
20-44	31(15.7)	11(7.5)					
45-64	71(36.0)	68(46.6)					
65-74	92(46.7)	66(45.2)					
75-84				81(84.4)	99(83.9)		
85 et +				15(15.6)	19(16.1)		
*Gender*							
Men	126(64.0)	100(68.5)		54(56.2)	83(70.3)		
Women	71(36.0)	46(31.5)		42(43.7)	35(29.7)		
*Primary rental disease*			NS			0.10	NS
Glomerulonephritis	38(19.3)	24(16.4)		9(9.4)	20(16.9)		
Diabetic nephropathy	45(22.8)	34(23.3)		19(19.8)	15(12.7)		
Cystic nephropathy	14(7.1)	17(11.6)		2(2.1)	2(1.7)		
Tubular-Interstitial nephropathy	19(9.6)	12(8.2)		4(4.2)	8(6.8)		
Vascular nephropathy	36(18.3)	20(13.7)		37(38.5)	51(43.2)		
Unknown	29(14.7)	25(17.1)		23(24.0)	15(12.7)		
Other	16(8.1)	14(9.6)		2(2.1)	7(5.9)		
*Renal biopsy*	43(21.8)	31(21.2)	NS	4(4.2)	14(11.9)	0.044*	0.047*
*Co-morbidities*							
Diabetes	65(33.0)	54(37.0)	NS	39(40.6)	34(28.8)	0.07	0.06
Chronic respiratory disease	15(7.6)	17(11.6)	NS	11(11.5)	14(11.9)	NS	NS
Heart failure	40(20.3)	28(19.2)	NS	42(43.7)	37(31.4)	0.06	NS
Cerebrovascular disease	10(5.1)	18(12.3)	0.016*	13(13.5)	16(13.6)	NS	0.09
Peripheral vascular disease	28(14.2)	37(25.3)	0.010*	26(27.1)	31(26.3)	NS	0.07
Arrhythmia	22(11.2)	31(21.2)	0.011*	29(30.2)	29(24.6)	^NS^	0.015
Coronary vascular disease	40(20.3)	33(22.6)	NS	35(36.5)	42(35.6)	NS	NS
Active malignancy	9(4.6)	12(8.2)	NS	7(7.3)	10(8.5)	NS	NS
*Risk factors*							
History of hypertension	148(75.1)	118(80.8)	NS	75(78.1)	101(85.6)	NS	NS
Smoker/Ex-smoker	71(36.0)	69(47.3)	NS	31(32.3)	45(38.1)	NS	NS
*Handicap*^*1*^	23(11.7)	19(13.0)	NS	6(6.2)	9(7.6)	NS	NS
*Mobility*			NS			0.002*	NS
Walk without help	169(88.5)	130(90.9)		57(63.3)	88(75.9)		
Need assistance for transfers	15(7.9)	6(4.2)		31(34.4)	18(15.5)		
Totally dependent for transfers	7(3.7)	7(4.9)		2(2.2)	10(8.6)		

P1. *P*-value <75 years (2002-2004 *vs* 2005-2007).

P2. *P*-value ≥75 years (2002-2004 *vs* 2005-2007).

P3. *P*-value Age x Period.

^1^including severe disability (vision impairment, paraplegia, hemiplegia, and amputation) and severe behaviour disorders (dementia, psychosis or severe neurosis).

*NS* Not Significant (with p>0.1).

**Table 2:** Biological baseline characteristics and conditions of dialysis initiation according to age and period

	**<75 yr**			**≥75 yr**			
	**2002-2004**	**2005-2007**	**P1**	**2002-2004**	**2005-2007**	**P2**	**P3**
	**(n = 197)**	**(n = 146)**		**(n = 96)**	**(n = 118)**		
*Serum albumin*			NS			0.027*	NS
<35 g/l	63(32.0)	51(34.9)		44(45.8)	50(42.4)		
≥35 g/l	78(39.6)	61(41.8)		21(21.9)	44(37.3)		
Missing	56(28.4)	34(23.3)		31(32.3)	24(20.3)		
*Body mass index*			NS			NS	NS
<18, 5 kg/m^2^	9(4.7)	5(3.6)		2(2.3)	7(6.1)		
18, 5–25 kg/m^2^	98(51.0)	67(47.9)		51(60.0)	64(56.1)		
≥18,5 kg/m^2^	85(44.3)	68(48.6)		32(37.6)	43(37.7)		
*Baseline eGFR*			NS			0.047*	0.08
<7 ml/min/1.73 m^2^	103(52,3)	59(40,4)		31(32,3)	46(39,0)		
7–10 ml/min/1.73 m^2^	47(23,9)	46(31,5)		26(27,1)	38(32,2)		
≥10 ml/min/1.73 m^2^	32(16.2)	32(21.9)		28(29.2)	31(26.3)		
Missing	15(7.6)	9(6.2)		11(11.5)	3(2.5)		
*Pre-dialysis anemia care*							
Hemoglobin			NS			<0.001*	0.005*
<11 g/dl	112(56.9)	77(52.7)		42(43.7)	74(62.7)		
≥11 g/dl	63(32.0)	57(39.0)		3637.5)	41(34.7)		
Missing	22(11.2)	12(8.2)		18(18.7)	3(2.5)		
Pre-dialysis ESA treatment	93(47.2)	85(57.8)	0.044*	51(53.1)	67(56.8)	^NS^	^NS^
*First treatment modality*			0.004*			^NS^	^NS^
Peritoneal dialysis	39(19.8)	17(11.6)		35(36.5)	32(27.1)		
Hemodialysis with fistula	77(39.1)	74(50.7)		18(18.7)	36(30.5)		
Hemodialysis without fistula	81(41.1)	55(37.7)		43(44.8)	50(42.4)		
In urgency	39(19.8)	25(17.1)	NS	16(16.7)	19(16.1)	NS	NS

P1. *P*-value <75 years (2002-2004 *vs* 2005-2007).

P2. *P*-value ≥75 years (2002-2004 *vs* 2005-2007).

P3. *P*-value Age x Period.

*eGFR* Estimated glomerular filtration rate, *ESA* erythropoietin-stimulating agent, *NS* Not Significant (with p>0.1).

When we assessed patients aged ≥75 years, we observed no significant difference in co-morbidities over time, although the proportions with diabetes, heart failure and arrhythmia decreased. The percentage of patients walking independently was higher in 2005–2007 than in 2002–2004 (p = 0.002). More biopsies were performed and more patients on dialysis had a serum albumin concentration ≥35 g/l during 2005–2007. The proportions of patients on dialysis with a baseline eGFR < 10 ml/min/1.73 m^2^ and with haemoglobin concentrations <11 g/dl were significantly higher during the second period.

The interaction between age and study period was tested for all baseline characteristics (p3 of Table 1). This interaction was significant for renal biopsy, arrhythmia, and haemoglobin concentrations.

### Patient survival

After 4 years of follow-up, 79 (82.3%) and 77 (65.2%) patients aged ≥75 years died in 2002–2004 and 2005–2007, respectively (p = 0.033). Only one underwent renal transplantation during follow-up. Among patients aged <75 years, 59 (29.9%) and 54 (37.0%) died within 4 years in 2002–2004 and 2005–2007, respectively. Fifty-eight patients (29.4%) underwent renal transplantation in 2002–2004 and 37 (25.3%) in 2005–2007 (p = 0.378).

**Figure 3:**
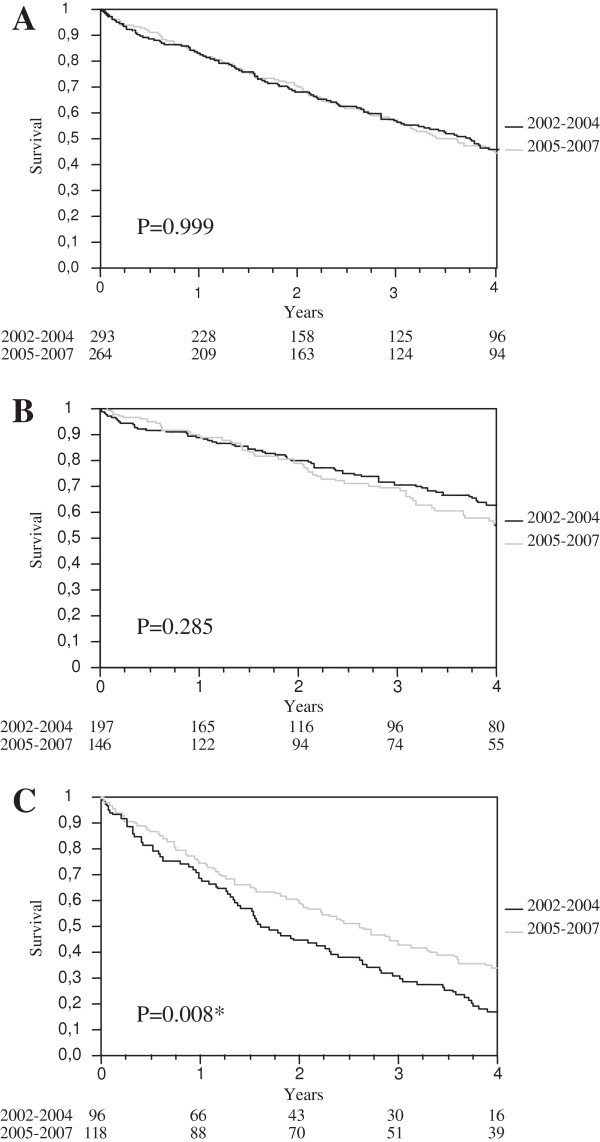
**Kaplan Meier analysis: 4-year survival according to the start of dialysis.** Legend of Figure 3: **A**. all patients (n = 557); **B**. <75 years (n = 343); **C**. ≥75 years (n = 214).

The Kaplan-Meier survival curves are shown in Figure 3. Median survival was stable in the entire cohort (3.7 years in 2002–2004 and 3.6 years in 2005–2007) and in patients <75 years old (p = 0.285). In patients aged ≥75 years, however, median survival was significantly longer in 2005–2007 than in 2002–2004 (2.6 *vs* 1.6 years, p = 0.008).

### Factors associated with survival in patients ≥75 years

As a survival difference was observed only for patients aged ≥75 years, we analysed factors potentially involved in improved survival of this group. Univariate analyses showed that period of dialysis initiation, primary kidney disease, diabetes, heart failure, impaired mobility, estimated glomerular filtration rate (eGFR) and first treatment modality were significantly associated with mortality.

Patients who started dialysis in 2005–2007 had a 19% lower risk of dying after 4 years (HR 0.81, CI 95% 0.7-0.9). Adjustment for co-morbidities resulted in a decreased risk of 15%, which remained statistically significant. There was no difference in fully adjusted mortality after introducing mobility (Model 3) or initial treatment condition (Model 4). Model 5 showed that heart failure, assistance needed for transfers, PD, and eGFR ≥10 ml/min/1.73 m^2^ were significantly associated with the better survival observed in 2005–2007 (Table 3).

**Table 3:** Adjusted hazard ratios of overall, 4-year mortality associated with study period (for all 214 patients over 75 years)

	**Model-1**			**Mdel-2**			**Model-3**			**Model-4**			**Model-5**		
	**Demographics**			**Demographics + Comorbidity**			**Demographics + Mobility**			**Demographics + Care**			**1 + 2 + 3 + 4**		
	**HR**	**95% CI**	**p**	**HR**	**95% CI**	**p**	**HR**	**95% CI**	**p**	**HR**	**95% CI**	**p**	**HR**	**95% CI**	**p**
*Dialysis initiation year. reference:*	*2002-2004*														
2005-2007	0.81	0.69-0.95	0.010*	0.85	0.72-0,999	0.049*	0.88	0.74-1.06	NS	0.85	0.72-1.00	0.052	0.94	0.78-1.13	NS
*Age. reference: 75–84 years*															
≥85 years	1.22	0.99-1.48	0.06	1.27	1.03-1.54	0.027*	1.18	0.95-1.44	NS	1.20	0.97-1.46	0.09	1.21	0.97-1.48	0.09
*Gender. reference: Male*															
Female	0.99	0.84-1.17	NS	1.00	0.84-1.18	NS	0.95	0.80-1.13	NS	0.97	0.81-1.15	NS	0.96	0.80-1.15	NS
*Comobidities. reference: without this comorbidity*															
Heart failure				1.31	1.11-1.55	0.001*							1.23	1.04-1.46	0.018*
Diabetes				1.21	1.02-1.43	0.030*							1.12	0.94-1.93	0.10
*Mobility. reference: walk out help*															
Need assistance for transfers							1.43	1.16-1.74	<0.001*				1.40	1.13-1.71	0.002*
Tatally dependent for transfers							1.42	0.97-1.96	0.07				1.38	0.94-1.93	0.10
*Dailysis modality. reference: HD with fistula*															
HD without fistula										1.29	1.03-1.62	0.025*	1.16	0.92-1.48	NS
PD										1.49	1.19-1.89	<0.001*	1.38	1.09-1.76	0.008*
*Baseline eGFR. reference: 7 ≥ eGFR < 10 ml/min/1.73 m*^*2*^															
eGFR < 7 ml/min/1.73 m^2^										1.08	0.88-1.33	NS	1.18	0.95-1.46	NS
eGFR ≥ 10 ml/min/1.73 m^2^										1.25	1.00-1.56	0.047*	1.36	1.08-1.71	0.009
Missing										1.13	0.79-1.55	NS	1.12	0.77-1.57	NS

*HD* hemodialysis, *PD* peritoneal dialysis, *eGFR* estimated glomerular filtration rate, *HR* hazard ratio, *95% CI* 95% confidence intervals.

Model-1 was adjusted for age and gender.

Model-2 was adjusted as Model-1 + heart failure and diabetes.

Model-3 was adjusted as Model-1 + mobility.

Model-4 was adjusted as Model-1 + dialysis modality and baseline eGFR.

Model-5 was adjusted for all variables in previous models.

*NS* Not Significant (with p > 0.1).

## Discussion

This study showed that the percentage of patients ≥75 years of age starting dialysis increased and that their initial clinical characteristics improved over time, improvements associated with significantly longer survival.

### Increases in elderly population

Over the past 10 years, the number of elderly patients starting dialysis has increased in many countries [1,6,10,16-18]. Our results confirm that the dialysed population in Limousin has increased in age. Several methods of calculation showed that 2002 remained an exception to the steady increase in the incidence of elderly persons starting dialysis, suggesting that the higher number of patients observed was probably due to sample fluctuations.

Many hypotheses have attempted to explain the significant increase in the incidence of elderly patients starting dialysis. The first hypothesis was an increase in the incidence of diabetes [4,5] and the improved survival from non renal diseases among people with chronic renal insufficiency [19,20]. Higher eGFR and wider access to dialysis were shown associated with the dramatic increases in incidence of older patients [18] and of patients of all ages [21] starting dialysis. We observed no difference in the proportion of patients ≥75 years with eGFR ≥10 ml/min/1.73 m^2^ in the two time periods, indicating that this factor cannot explain the increase in ESRD incidence among elderly people in Limousin. Improved patient care, including early referral to nephrologists prior to dialysis, may be associated with the increased incidence of elderly subjects starting dialysis, by reducing death before ESRD. Indeed, the increased proportions of our patients with fistula before dialysis and better clinical status strengthen this hypothesis. Improvements in pre-dialysis monitoring can lead not only to patients starting dialysis at a later age, but also to improvements in their general condition and a lower eGFR before starting dialysis. Presumably, some patients who would not have survived before reaching ESRD would now survive and be candidates for dialysis.

The urgency of the first dialysis did not differ between the two time periods, regardless of age, and did not affect survival. This variable therefore did not provide information on nephrological follow-up prior to the start of dialysis.

### Progression over time

We also observed changes in clinical status and patient care between the two periods. The percentage of elderly patients undergoing renal biopsies increased significantly, resulting in a decreased in the rate of patients with indeterminate renal diseases and indicating that the management of this population changed before the start of dialysis. There was a correlation between age and the start of dialysis, indicating an increase in work-ups at the beginning of dialysis.

Disability among older patients was modified over time, with older patients having improved autonomy and the percentage walking without help increasing significantly. Although nutritional status may difficult to determine only from measurements of serum albumin concentration, the rate of albuminemia decreased, suggesting improvements in nutritional status and/or inflammatory responses. This finding is consistent with previous results, which underlined the importance of monitoring albumin concentration [22]. In contrast, pre-dialysis anemia care became worse for patients over time, a finding consistent with international recommendations of a lower target haemoglobin concentration, resulting from findings suggesting that administration of high EPO does not reduce mortality or cardiovascular risk prior to the initiation of dialysis [23,24].

Although a higher BMI is associated with worsening co-morbidities in younger Caucasian populations, this was less evident in older patients [25]. In our study, this was difficult to determine, inasmuch as only 2 patients ≥75 years old had a BMI <18.5 kg/m^2^ in 2002–2004.

The increase in the percentage of patients undergoing initial haemodialysis with fistula was partly due to a decrease in haemodialysis without fistula, but was also to a decrease in first-line treatment of patients by PD. This difference was significant only for patients <75 years old.

### Survival

The main finding of our study was the improvement in survival of elderly patients over time. In contrast, there were no significant differences in the entire cohort or the younger patient group between the two periods. Age has been reported to influence survival in dialysed elderly patients [7,18,26], although other studies [10,11,27] have found that age was not an independent predictor of survival in elderly patients. A method of predicting 6-month prognosis in elderly patients starting dialysis found that functional limitations were better predictors than age [27]. When this score was applied to our cohort, the mortality rates were similar to those presented, at least for groups containing large numbers of patients (data not shown). Thus, our results were in agreement with studies showing that neither age nor gender was significantly associated with survival among elderly patients. Moreover, our findings emphasize the importance of functional limitations in predicting survival. Improved autonomy, including being able to walk unaided, was significantly associated with the increase in survival over time. Walking has already been found to be strongly associated with mortality in aged populations, a relationship not altered by dialysis [17,28].

The impact of the method of dialysis on survival is unclear. Although studies have analysed the link between dialysis methods and survival [17,26,29], those studies were criticized due to the selection bias inherent in the choice of dialysis method, which could explain apparent differences in mortality rate [30]. Indeed, peritoneal dialysis is often promoted for patients with diabetes [31], elderly patients [32] and patients with heart failure [33]. Although this requires further investigation, evaluating the impact of dialysis method on survival is very difficult, with the method adjusted for individual patients.

The main limitation of this study was its sample size. Although Limousin has the highest proportion of people over 75 years old in France, it is one of the least populated regions. Thus, some variables with borderline significance may be more sensitive to adjustments. Another limitation was that our study included only ESRD patients starting dialysis, excluding a small number of patients with serious ESRD who were treated conservatively and introducing a selection bias. Following the evolution of this population over time seems to be important. As in many regions of France, a system monitoring early renal failure was recently initiated in Limousin. Nephrological follow up before dialysis has an impact on quality of life. In the elderly, the quality of life is better when the first dialysis has been programmed [34]. Finally, we compared patient survival with data at the initiation of dialysis. Changes in patient co-morbidities and their care after dialysis initiation were not considered.

This study also has several strengths. The small number of nephrologists and their involvement during the pilot phase of the registry ensured stability in coding and completeness and resulted in rigorous patient follow up. Thus, our cohort was not subject to the bias frequently observed in larger cohorts, due to the exclusion of patients with missing data or difficult compilation from various sources of information.

## Conclusions

In conclusion, we observe an increase over time in the incidence of elderly patients starting dialysis, as well as a decrease in the differences between younger and elderly patients. Both of these findings may be due to global improvements in the health status of elderly individuals. The decrease in some co-morbidities and improvements in functional limitations were associated with significantly improved survival among patients ≥75 years. Our findings suggest that older individuals are better managed during the progression of chronic kidney disease, a finding requiring confirmed in other cohorts of patients with chronic kidney disease.

## Competing interests

The authors declare that they have no competing interests.

## Authors’ contributions

FG conceived of the study, performed the statistical analysis and drafted the manuscript. CH, JA, VA, FB, RB, BC, JPC, MC, ZD, ME, PH, CL, CL, MM, PP, JMP, JPR, and MR checked and validated data and helped to draft the manuscript. CC participated in the statistical analysis and helped to draft the manuscript. JCA participated in its design and coordination and helped to draft the manuscript. All authors read and approved the final manuscript.

## Pre-publication history

The pre-publication history for this paper can be accessed here:

http://www.biomedcentral.com/1471-2369/14/131/prepub
